# Building leadership capacity among junior faculty: Evaluating multi-level outcomes of a leadership program

**DOI:** 10.1017/cts.2023.529

**Published:** 2023-04-13

**Authors:** Layla Fattah, Lisa Bloom, Cara Della Ventura, Janice Gabrilove

**Affiliations:** 1 ConduITS, Institute of Clinical and Translational Sciences, Icahn School of Medicine at Mount Sinai, New York, NY, USA; 2 Graduate School of Biomedical Sciences, Icahn School of Medicine at Mount Sinai, New York, NY, USA; 3 Talent Development & Learning, Mount Sinai Health System, New York, NY, USA

**Keywords:** Leadership, junior faculty, team science, organizational leadership, evaluation

## Abstract

**Introduction:**

Leadership is recognized as an essential competency across healthcare and science. The LEAD (Leadership Emerging in Academic Departments) program at the Icahn School of Medicine at Mount Sinai (ISMMS) is a structured 12-month blended learning program that catalyzes personal and professional leadership skills, behaviors, and capacity.

**Methods:**

Utilizing a post-program survey design, the Leadership Program Outcome Measure (LPOM) explored self-reported impact of the LEAD program on leadership knowledge and skills in relation to personal and organizational leadership constructs. Application of leadership skills to practice was tracked via completion of a leadership-focused capstone project.

**Results:**

Over 3 cohorts, 76 participants graduated and 50 completed the LPOM survey (68% response rate). Participants self-reported an increase in leadership skills, conveyed plans to use acquired skills in current and future leadership positions, and noted improved leadership skills across the personal and organizational domains. Comparatively less change was detected at the community level. Tracking of capstone projects found that 64% of participants were able to successfully implement their project in practice.

**Conclusion:**

LEAD was successful in promoting the development of personal and organizational leadership practices. The LPOM evaluation provided a valuable lens through which to assess the individual, interpersonal, and organizational impact of a multidimensional leadership training program.

## Introduction

Leadership is increasingly recognized as core competency to advance individual and collective goals across healthcare and science, and a critical element in competency-based education for clinical and translational science (CTS) [1]. A national survey of North American Academic Health Centers found 99% of respondents (58% response rate) provided some form of leadership development training [2]. These leadership programs, however, often lacked a theoretical framework of leadership to guide educational content[4] that enhances self-awareness, interpersonal effectiveness, team management, and change management as a set of competencies that advance leadership capacity. Skills related to these competencies are critical for junior faculty investigators engaged in clinical and translational research, where a core aim is to work collaboratively across disciplines with a multitude of stakeholders to solve complex problems in medicine designed to improve human health. A gap exists in meeting the specific learning needs of junior faculty investigators, who require leadership acumen to advance their careers and engage in meaningful science and team science. In addition, the majority of existing programs fail to measure outcomes that demonstrate program effectiveness [2], partly due to a lack of program evaluation of leadership-related outcomes.

The Icahn School of Medicine at Mount Sinai (ISMMS) has responded to this unmet need to assure leadership readiness for the CTS Junior Faculty workforce, including instructors, assistant professors and early (within the first year) associate professors, to advance leadership capacity through the design, development, implementation, evaluation, and dissemination of a Leadership Emerging in Academic Departments (LEAD) Program.

We conducted a post-program online survey, administered to each cohort as they graduated from the program to assess participant’s perceived attitude and behavior change in the domains of individual leadership, organizational leadership, and community leadership[3]. This was followed by an online survey in 2020 with graduates from 2017 to 2020 to explore participants’ perceived downstream behavioral change, including individual growth. We also report on LEAD Program Capstone projects completed by LEAD participants, designed to operationalize knowledge of leadership principles and encourage participants to integrate theoretical leadership constructs into research practice. This article describes the development, delivery, and evaluation of LEAD program for junior faculty at the ISMMS.

## Methods

### Overview of the LEAD Program

The LEAD Program was designed to deliver a structured 12-month blended learning experience for junior faculty. The design of our LEAD program was initially informed by a leadership program developed at the University of Texas Southwestern Center for Translational Medicine. Our subsequent adapted program was specifically customized to our cohort of learners, using a consultative approach to learning [4], whereby stakeholders were engaged in developing a bespoke curriculum. We incorporated educational content to address perceived leadership characteristics and success factors that are critical to the development of junior faculty investigators. Such content included strategic thinking, managing conflict and challenging conversations, change management, emotional intelligence, team building, business planning, and personal leadership development [2]. The overarching goal of the LEAD program was to (1) enhance personal and professional leadership capacity, skills, and behavior; (2) enable participants to systematize their intuitive leadership skills in a structured and supportive environment and to capitalize on their strengths and recognize their developmental opportunities, (3) apply leadership skills to solve real-world practice-based challenges, and (4) provide a platform for fostering interprofessional and cross-disciplinary relationships among junior faculty from a range of scientific and clinical backgrounds to create a community of practice. Underlying the program is a learning framework to foster behaviors, attitudes, mindsets, and strategies that translate learning into effective role performance and organizational outcomes. This LEAD program was comprised of an asynchronously and synchronously delivered curriculum, personal and group coaching, and a capstone project to apply theory to practice.

### LEAD Program Education Faculty and Professional Development Educator Team

The LEAD program sought to leverage diverse leadership training and professional development expertise through collaborative engagement with content experts in both the Icahn School of Medicine at Mount Sinai (ISMMS) and the Mount Sinai Health System (MSHS), including the Office of Academic Development and Enrichment (OADE) and Talent Development and Learning (TDL). To further capitalize on the wealth of institutional knowledge to advance leadership competency, program faculty were recruited from the Institute of Medical Education, the Office of Human Resources, The Center for Multi-Cultural and Community Affairs, and the Office of Diversity and Inclusion and drew upon the informed perspectives of senior leadership including the Chief Transformation Officer for the MSHS, the Deans for the medical school and scientific affairs, respectively, as well as Presidents of the Mount Sinai Hospital, Mount Sinai Queens, Mount Sinai West, and Mount Sinai Downtown. In addition, we partnered with external consultants that brought expertise not readily available within the organization, including business leaders, and leadership faculty.

### LEAD Program Participant Recruitment

We designed an RFA and solicited applications from junior faculty – instructor through first-year associate professor. Applicants were asked to provide an overview of their experience in formal or informal leadership positions, their most significant achievements in clinical care, CTS research, teaching, and community engagements, as well as their reasons for wanting to participate in the LEAD program. Applicants were also asked to provide demographic information, a CV or biosketch, and a letter of support from their Department Chair, Institute or Center Director. Two program leaders reviewed applications, and applicants were scored using a rubric that ranked previous leadership experience, career stage, evidence of Department Chair, Institute or Center Director support, motivation to participate, and overall quality of the application. In addition, the program sought to gather representation from a diversity of scientific/medical disciplines, degrees, departments, and MSHS sites.

### Curriculum

The LEAD Program is built on a constructivist model [5], whereby participants are lead through a process of self-reflection and discovery, skill acquisition, behavioral development, and coaching to embrace foundational principles fundamental to academic leadership. This approach incorporates best practices for leadership development, including the use of experiential activities to advance capabilities and opportunities for self-reflection and personal growth [6,7].

**Fig. 1.**
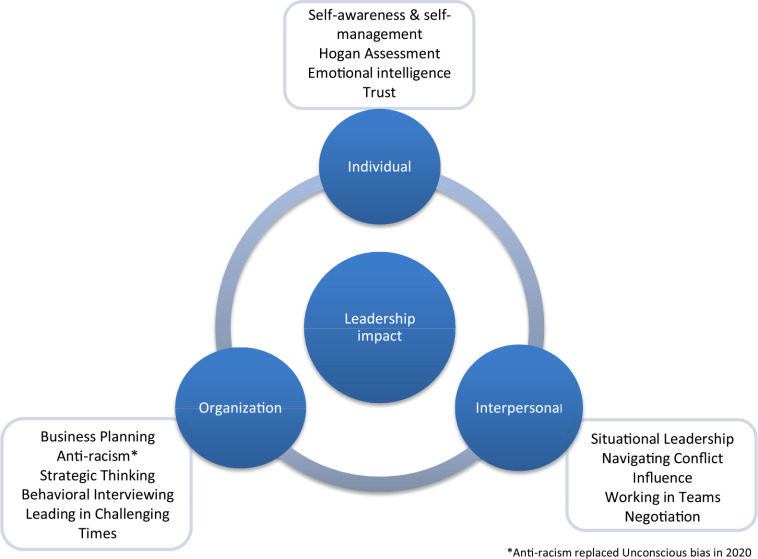
LEAD conceptual curricular framework.

The program is comprised of 12 interactive competency-based workshops, each focused on a specific leadership skill or attribute. To foster self-development, each participant completed the Hogan Leadership Forecast Series [8], the Emotional Quotient Inventory (EQ-i 2.0) [9], and the Thomas-Kilmann Conflict Mode Instrument [10]. These tools are utilized to promote self-awareness of participants’ leadership tendencies and styles. Workshop topics flow through blocks of learning that focus first on individual leadership, then on interpersonal leadership (leadership constructs governing interactions with others), and, lastly, on organizational leadership, which includes strategic thinking and business planning. A visual representation of the curriculum-mapped topics is delineated in Fig. 1. Using a blended learning approach, online videos and resources were provided in a virtual learning platform to establish the context and provide relevant tools for the subsequent workshops. Monthly, synchronous workshops were used for application of learning through case studies, inquiry-based learning, peer discussion, and role play. Each workshop was four hours long, with a break for lunch to facilitate networking and informal discussion. Participants were encouraged to maintain a journal thereby enabling them to focus on self-reflection and goal setting to advance experiential application of new knowledge and skills. Participant satisfaction was gathered via a satisfaction survey administered at the end of each workshop. Feedback was reviewed on an ongoing basis and used to facilitate program development and improvement.

### Leaders’ Labs, Coaching, and Support

The LEAD workshops were supplemented by one-on-one assessment debriefs, one-to-one coaching (as requested), and an optional “Leaders’ Lab,” a monthly faculty-facilitated peer coaching session. The Hogan and EQ-i 2.0 assessment debriefs served to increase participants’ self-awareness and enabled them to establish self-development goals for the program. Upon request, the debriefs continued with one-on-one coaching to address individual development needs. The Leaders’ Lab was an optional peer-to-peer coaching that was designed to support the transfer of learning to the workplace. Through this, the participants were able to meet monthly in small groups of 6**–**10 participants. The 60- to 90-minute meetings were facilitated by a TDL Coach and were designed to foster a safe, trusting, and confidential dialogue. This coaching opportunity offered participants a sounding board for leadership challenges, encouraged them to reflect and learn from peers, created a space for sharing lessons learned and best practices, and provided them with support in navigating the health system. It also helped them to build/expand their internal network and develop a sense of community. Almost all participants attended the Leaders’ Lab sessions, and approximately five participants per cohort took advantage of the one-to-one coaching.

### The LEAD Program Capstone Project

Participants were encouraged to identify a problem or issue within their own sphere of practice that could be addressed through the use of leadership concepts and newly acquired leadership skills. The final output of the program was a Leadership Capstone project, which was designed to integrate leadership principles from the curriculum in order to solve a practical issue, thereby demonstrating competence and reinforcing application of learning to practice [4]. The aim was for participants to conceptualize the capstone project during the LEAD Program. Participants submitted a project abstract mid-year and received feedback from program leadership on the suitability of their proposed capstone project as it related to incorporation and application of leadership concepts.

**Fig. 2.**
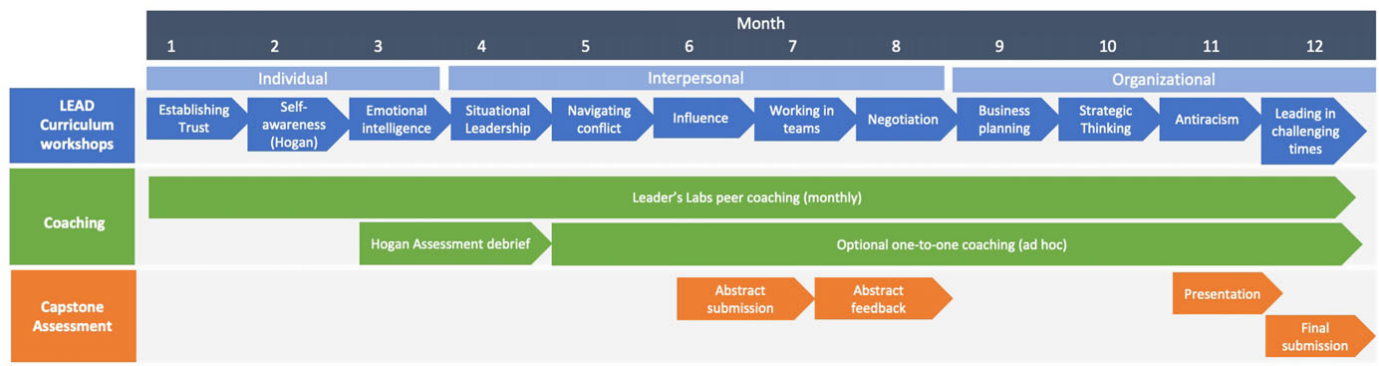
LEAD curriculum timeline.

At the end of the program, the final capstone report was evaluated using a standardized, bespoke assessment rubric that considered the project focus, evidence of leadership impact, action planning and implementation, and outcome measures and metrics of success employed. Participants were then provided with an opportunity to make additional revisions to their written report based upon formative feedback. Lastly, participants partook in an end-of-the-year event which included a formal presentation of their project to their peers and faculty. Participants received feedback from leadership and peers during their presentation and had the opportunity to edit their projects prior to final submission. The capstone presentations were followed by a celebratory event acknowledging the accomplishments of participants and serving as a formal end to this phase of the program. An overview of the full curriculum timeline is presented in Fig. 2.

## Evaluation

### Assessment of Leadership Competency Attainment

#### Leadership Program Outcomes Measure (LPOM)

A validated post-program data collection tool, the Leadership Program Outcomes Measure tool (LPOM) [3], was adapted with permission from the original authors for use with each of the cohorts in this program. The LPOM uses a post-program survey design utilizing a degree-of-change approach. This rating measures the amount of change better than evaluations that measure change using pretest and post-test ratings which can be subject to response-shift bias. Response-shift bias occurs when individuals have rated themselves at one time, from one perspective, and then change their responses later as their perspectives change [11]. This issue is pertinent in leadership, where participants may have limited knowledge at the beginning of a program, which prevents them from accurately determining their baseline behaviors. Response-shift bias is avoided when participants rate themselves within a single frame of reference [12]. In reviewing this tool, the focus of survey items was pertinent to the overarching objectives of the LEAD program. Some minor wording adaptions were made to ensure the survey items were clearly understood by LEAD program participants, and a number of non-relevant items were excluded. The revised instrument was checked for face and content validity by a panel of faculty and administrators familiar with the program.

The survey uses a mixed-methods approach and includes 23 Likert-type scale questions that ask the participants to rate the perceived impact of the program as such impact relates to the programmatic goals of individual leadership (confidence, growth mindset), organizational leadership (networking, problem-solving, innovation), community leadership, referring to the larger professional community (cultural competency and expanded spheres of influence). Each section also provided an open-ended question intended to capture respondents’ experiences in their own words and to allow for triangulation with Likert scale responses to better understand quantitative data. An additional open-ended question aimed to solicit any further feedback on the LEAD experience and/or on other dimensions of change not addressed in the questioning. The survey concluded with three 10-point scale questions asking about the importance and impact of LEAD overall.

The survey was created in RedCAP, and each cohort of LEAD participants was invited to complete the survey via email at the end of their program of study. Two reminder emails spaced two weeks apart were sent to encourage participation from non-respondents. Overall response rate was 68%.

Quantitative data from the LPOM were analyzed using IBM SPSS Statistics to compute means, standard deviations, and percentages. The 5-point Likert scale items were assigned numerical values (not at all = 1; a great deal = 5) and were treated as interval data. In line with best practices, in addition to reporting mean values for each item, we also reported responses as frequencies (percentages of responses in each category) [13]. For open-ended qualitative items, the program team manually organized and coded the data. Author team members independently reviewed the qualitative responses, developed initial codes, and formulated themes. Through iterative discussion, a final set of themes and subthemes was generated. We then integrated quantitative findings with emergent qualitative themes.

## Results

### LEAD Participant Characteristics

Between 2017 and 2020, the LEAD graduated three cohorts with a total of 74 participants (Table 1). Represented in the [cohorts] were a diversity of degrees, including MD (55), MD/PhD (9), MD/MPH (9), MD/MSCR (2), MD/MBA (1), MD/Diplomat in Clinical Informatics (1) PhD (8) PharmD (1) and Nurse practitioner (NP) (1), and several medical and scientific disciplines that represented 24 Basic Science and Clinical Departments across eight sites within the MSHS. Among the participants, 59.5% were women and 22% were from racial or ethnic groups underrepresented in science and medicine (URiSM). They provided training for junior faculty at both the assistant and early associate professor rank, respectively.

**Table 1.** Participant demographics 2017-2020

Cohorts	2017–18 cohort 24 participants	2018–2019 cohort 24 participants	2019–2020 cohort 26 participants	Overall 74 participants
Response rate to survey	20 (83%)	16 (67%)	14 (54%)	50 (68%)
Male	11 (46%)	9 (37.5%)	10 (38%)	30 (40.5%)
Female	13 (54%)	15 (62.5%)	16 (62%)	44 (59.5%)
Non-underrepresented	14 (58%)	18 (75%)	19 (73%)	51 (69%)
Underrepresented in Science and Medicine (URiSM)	7 (29%)	6 (25%)	3 (12%)	16 (22%)
Prefer not to answer	3 (13%)	0 (0%)	4 (15%)	7 (9%)
Assistant Professor	14 (58%)	18 (75%)	19 (73%)	51 (69%)
Associate Professor	10 (42%)	6 (25%)	7 (27%)	23 (31%)
MD	15 (63%)	20 (83%)	20 (77%)	55 (74%)
MD/PhD	4 (17%)	2 (8%)	3 (12%)	9 (12%)
PhD	4 (17%)	2 (8%)	2 (8%)	8 (11%)
Other clinical degree (e.g. PharmD, NP)	1 (3%)	0 (0%)	1 (4%)	2 (3%)

### Capstone Projects

Over the three program cohorts, participants completed projects across a wide variety of self-reported areas including quality and process improvements (51%), new clinical programs (24%), business plans (10%), new research programs (9%), and new educational initiatives (7%). A number of important results were achieved including improvements in quality of care, patient safety, and efficiency of care processes; enhanced patient satisfaction; and new program development. Submitted capstone projects were marked using a template marking scheme, and formative feedback was provided. Fifty-two percent of the participants across all cohorts had implemented their capstone projects upon completion of their LEAD program.

Participants who had not implemented their capstone projects in practice were asked on a scale of 1–10 how likely they were to do so. The mean response was 8.92. Finally, participants were asked for an overall rating of how important it was to them to implement the learning from LEAD in their practice. The mean response was 8.87.

### Evaluation

#### LPOM Outcomes

The LEAD program was perceived by participants to generate impact at multiple levels. The results of the qualitative and quantitative analysis are integrated below under the survey themes of (1) individual, (2) organizational, and (3) community levels.

#### Theme 1: Individual-Level Outcomes (Table 2)

As result of LEAD, a majority indicated noticeable changes in individual leadership skills, attitudes, and/or behaviors related to leadership. Specifically, the most commonly endorsed individual changes include a positive effect on personal growth (4.64), increased confidence in handling leadership roles or responsibilities (3.47), increased empowerment to make a difference through leadership (4.11), and improved creative thinking (3.47). Post-completion of LEAD, participants also reported an increase in the awareness of the value of their time and connection to leadership role models (4.27).

**Table 2.** Individual-level outcomes

	Not at all (%)	Not very much (%)	Some (%)	Much (%)	A great deal (%)	Mean response	Standard deviation
I improved in self-confidence in handling leadership roles/responsibilities	2.2	8.9	44.4	28.9	15.6	**3.5**	0.93
I improved in creative thinking	2.2	11.1	40.0	31.1	15.6	**3.5**	0.96
I improved my leadership skills	2.2	8.9	31.1	35.6	22.2	**3.7**	0.99
I was able to meet people whose success I could imitate	0	2.2	15.6	35.6	46.7	**4.3**	0.80
I increased my awareness of the value of my time	0	4.4	17.8	37.8	40.0	**4.1**	0.86
Exposure to new ideas and concepts during LEAD led to my personal leadership growth	0	0	4.6	27.3	68.2	**4.6**	0.57
Through LEAD I felt empowered to make a difference through leadership	0	4.6	13.6	47.7	34.1	**4.1**	0.80

Key themes from the qualitative individual-level outcome data indicated primary changes to self-confidence and self-awareness.

In terms of confidence, one participant stated: “I gained the confidence to take my goals, both research and clinical, to the next level in developing my vision into programs that could be implemented hospital-wide. I did not have the personal confidence or the leadership acumen to begin to do this prior to the LEAD program.”

In terms of self-awareness, participants highlighted the value of the Hogan assessment in promoting insight into their own behavioral tendencies and motives/values/preferences. One participant stated, “I have become more self-aware. I understand my strengths and weaknesses especially pertaining to leadership style.”

Related to empowerment, one participant said “I think about leadership different[ly] and now see myself as a budding leader when I didn’t before. The program helped me to think about the varied ways that I can display leadership skills and that leadership is a skill that can be developed and learned.”

#### Theme 2: Organizational Level (Table 3)

In terms of organization leadership, participants described how participation in the program led to wider networks of leadership contacts that transcended departmental boundaries (4.14), improved ability to network (3.58), and an ability to leverage connections to facilitate change in their own leadership practice (4.23). Participants indicated an increased ability to respond to leadership problems and situations effectively (3.89) and be more innovative in their approach to solving problems (3.7).

**Table 3.** Organizational-level outcomes

	Not at all (%)	Not very much (%)	Some (%)	Much (%)	A great deal (%)	Mean	Std Deviation
I improved my organizational decision-making skills	0	8.9	42.2	35.6	13.3	**3.5**	0.83
I improved my networking skills	0	11.1	35.6	37.8	15.6	**4.0**	0.88
I am able to respond to problems and situations more effectively	0	4.4	22.2	53.3	20.0	**3.9**	0.77
I became more innovative in my approach to problem-solving	0	6.8	25.0	43.2	25.0	**3.7**	0.87
I learned to make more efficient use of my time	2.3	11.4	43.2	34.1	9.1	**3.4**	0.88
The exposure to other people and ideas helped facilitate change in my practice	0	2.3	9.1	52.3	36.4	**4.2**	0.70
I became more involved in leadership projects	2.3	11.4	31.8	31.8	22.7	**3.6**	1.03
I became more efficient in my use of resources	0.0	6.8	36.4	38.6	18.1	**3.7**	0.85
I developed the confidence to compete on a different level in career	2.3	13.6	29.6	27.3	27.3	**3.6**	1.09
LEAD helped me to build a better network of leadership contacts outside my own department	0	9.1	11.4	36.4	43.2	**4.1**	0.94

The qualitative data from respondents described the LEAD program as a means of providing connection to organizational leadership. One participant indicated, “Since we were introduced to the hospital presidents, department chairs, and CMOs, I was better equipped to manage those conversations when I met my own hospital president to request for resource allocation.”

Other graduates described the benefits of sharing perspectives with individuals from other departments or disciplines. One participant stated, “the most durable and beneficial effect of the program [is the] interaction with professionals from other departments and sites in the MSHS which offers new perspectives.”

Participants also increased their understanding of MSHS as an organization and credited the LEAD program as increasing their ability to navigate the organizational landscape. As one participant stated, “I am more open to the idea of taking on a leadership role within my department; I feel better equipped to take on this kind of role because I understand the organizational structure of the hospital/medical center a lot better now.”

#### Theme 3: Impacts at Community Level (Table 4)

We considered community level to be both the MSHS community and the wider medical/scientific community beyond the organization. Participants were able to increase their involvement in other areas of MSHS (3.21) but little change was reported in leadership involvement outside MSHS at the regional (2.20), national (2.23), and international levels (1.81).

**Table 4.** Community-level outcomes

	Not at all (%)	Not very much (%)	Some (%)	Much (%)	A great deal (%)	Mean	Std Deviation
Due to my LEAD participation I increased my involvement with regional organizations	36.4	25.0	27.3	4.6	6.8	**2.2**	1.18
Due to LEAD I became involved with groups on a national level	40.9	20.5	20.5	11.4	6.8	**2.2**	1.28
Due to LEAD I became involved with activities at an international level	60.5	14.0	16.3	2.3	7.0	**1.8**	1.21
My LEAD experience helped to increase my involvement in other areas of MSHS	9.3	18.6	30.2	25.6	16.3	**3.2**	1.19
I reduced my commitment to some activities to be more effective in other aspects of my work	11.6	16.3	30.2	28.0	14.0	**3.2**	1.20
My appreciation of cultural differences increased due to my LEAD participation	7.0	14.0	25.6	37.2	16.3	**3.4**	1.13

From the qualitative data, it appeared it was too soon for some to explore the impact of LEAD on participant’s involvement in the wider community. As one participant commented, “Although I have gained an appreciation for the importance of national and international community participation as part of the LEAD program, during the course of the program I have not yet engaged in these efforts. However, I have made it a personal goal to do so in the upcoming year.”

A smaller number of participants were able to provide concrete examples of wider community involvement. One participant shared “1. I became treasurer of a medical society 2. I became involved in organizing two international scientific conferences. 3. I became an associate scientific advisor for a major journal. 4. I was selected as reviewer for my first NIH study section.”

## Discussion

The LEAD was created, implemented, and evaluated to advance competency-based leadership capacity among junior faculty clinical and translational science investigators. The successful attainment of leadership competencies was evaluated both subjectively, the validated and adopted LPOM tool, designed to assess the self-reported impact of this training on leadership capability by participants, and objectively via the successful completion of a rubric-assessed capstone project. Utilizing the LPOM, participants reported an increase in self-assessed leadership skills, conveyed plans to use acquired skills in current and future leadership positions, and noted improved leadership skills across the personal and domains. In addition, participants demonstrated effective integration of leadership concepts and through the development and successful implementation of a wide range of leadership capstone projects. Subsequent tracking (1**–**3 years post) of capstone projects found that 64% of participants were able to successfully implement their project in practice.

Data from the LPOM survey demonstrated self-reported positive changes in self-efficacy and self-confidence with regard to leadership skills at individual and organizational levels. At the individual level, reported outcomes indicated positive changes in personal growth, self-confidence, exposure to new ideas and concepts, and relationship development. In particular, the construct of self-confidence appears multiple times, which is widely considered to be an essential characteristic for effective leadership [14]. We believe providing the opportunity to interact with leadership role models through the program, an option that is not always freely open to junior faculty, is formative in underpinning these positive changes.

At the organizational level, participants reported improvement in networking, management skills (e.g. decision-making and problem-solving), and knowledge of the MSHS organizational structure. Participants largely attributed these changes to their facilitated interactions with senior leadership at Mount Sinai, for example, hospital presidents and chairs. Participants engaged in facilitated Q&A sessions with these leaders, which provided the opportunity for them to make new connections, practice their communication skills with senior leaders, and gain a better understanding of the extremely complex matrix organizational structure at MSHS. We also have seen the development of a strong support network between participants, particularly through the Leaders’ Lab, resulting in a community of practice that has extended beyond the program.

Comparatively less change was detected at the community level. It may be that change in this domain takes longer to implement and may be more challenging as it is linked with a shift in organizational culture to further advance the cross-collaboration that requires a breakdown of established silos.

The existing organizational culture, with its established systems and processes, may impact participants’ ability to implement their capstone project. Although a small majority of the LEAD cohort were able to implement their capstone projects during the LEAD program (one participant was awarded a FOJP Innovation Grant for his capstone work), the remaining participants did not implement their projects although they showed a strong desire to do so following completion of the LEAD program. We hypothesize that time and workload constraints may be another barrier to successful project implementation during the LEAD program.

To address the lack of change at the community level, we recognize a need to support LEAD participants in their efforts to extend their influence to the wider professional community both within and beyond MSHS. Evidence suggests that, in general, the culture of an organization affects the degree to which learned behavior will be transferred to practice [15]. Participants themselves recognized the need for ongoing leadership learning and application of that learning to practice. In 2020, a group of program alumni established the LEAD Ambassador Program to foster a culture of leadership learning. This program brings together former LEAD participants on a regular basis to maintain motivation, share challenges and successes, form an ongoing community of practice that provides peer support and coaching, and extend mentorship to junior faculty throughout MSHS.

### Limitations

In discussing the importance (and benefit) of measuring leadership change at the individual, organizational, and community levels, we also recognize the limitations of using self-reported data as a substitute for more objective observational data. Participants may provide an underestimation or overestimation of the knowledge and skills gained with the latter being more likely because of the potential for social desirability bias and effort justification bias. We also must highlight the difficulty in accessing observational data in this context. Furthermore, only focusing on these participant-level, self-reported outcomes neglects the impact these leaders have on their subordinates, peers, and superiors [16].

### Future Work

The LPOM provided us with an in-depth self-assessment of change following the LEAD program. In the future, we intend to also track objective measures of productivity, including promotions, new leadership roles, and publications based on capstone projects. In addition, emerging data suggest that leadership training contributes to resilience and we intend to more rigorously assess this contribution in future iterations of the program. Future plans for the LEAD program involve including a 360-degree assessment process, allowing participants to gather data from peers and colleagues to provide an external perspective of change. Furthermore, the launch of the Ambassador program provides a further opportunity to advance leadership capacity though continued learning with peers.

## Conclusion

LPOM has provided a lens through which to assess the individual, organizational, and community impact of a multidimensional leadership training program. LEAD provides an educational initiative that promotes the development of personal and organizational leadership practices. This is important, given the overwhelming need for leadership skills to promote early career faculty in meeting the demands of advancing science to meet the complex demands of translational science. We have presented findings from three cohorts of participants; future research will evaluate the longer-term impact of the LEAD program, especially with regard to the development of an alumni program and an ongoing approach to the leadership development at the MSHS.
